# Interactions of Water Molecules Adsorbed on Hydrophilic Substrates Clarified by Transport Characteristics of Graphene

**DOI:** 10.1021/acsomega.6c05969

**Published:** 2026-09-08

**Authors:** Yi Chen, Misuzu Kitahara, Lei Zhi, Yoshihiro Kubozono, Hidenori Goto

**Affiliations:** Research Institute for Interdisciplinary Science, 12997 Okayama University, Okayama 700-8530, Japan

## Abstract

Hydrophobic treatment of SiO_2_/Si substrates is indispensable for achieving excellent transport characteristics in graphene devices. Although the interaction of the carriers with electric dipole moments of water molecules is believed to degrade the transport characteristics, the configuration and electric-field-induced control of molecular alignment remain to be elucidated. In this study, we investigate the electric polarization of the water molecules adsorbed on hydrophilic SiO_2_ by measuring the conductivity of graphene field-effect transistors (FETs). The conductivity is found to be highly sensitive to polarization-induced changes in carrier accumulation. Consequently, we demonstrate that carrier accumulation can be enhanced by interfacial water at room temperature. Furthermore, the temperature and gate voltage dependences of the conductivity reveal the phase diagram of the interfacial water/ice layer. Notably, the freezing point is suppressed at zero gate voltage due to the electric field induced by negative charges on the SiO_2_, whereas it is enhanced with increasing gate voltage. The stable ice phase without electric polarization is also observed at high gate voltages.

## Graphical abstract

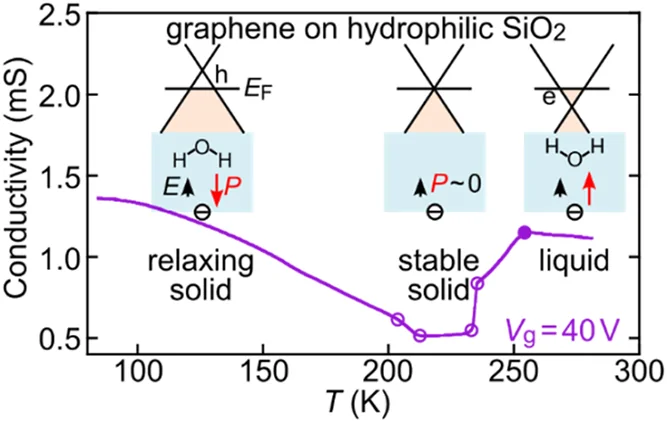

## Introduction

The structure and phase behavior of water confined in nanoscale spaces have been important topics in biological systems, condensed matter physics, nanoscience, and interface science.^1–4^ Confined water exhibits distinct molecular arrangement, hydrogen bond networks, and phase behaviors owing to interfacial interactions at spatial boundaries, as well as intermolecular interactions. Consequently, confined water displays unique physical properties, such as altered transition temperatures and ice structures, which depend on the confinement gap size and the hydrophilicity of the boundary.^5–7^ These various phenomena arise from directional hydrogen bonds that characterize the structure in the solid phase. In conventional ice phase Ih, the orientation of hydrogen bonds is random; thus, even though individual water molecules possess an electric dipole moment (EDM), the net electric polarization vanishes due to random molecular orientations. Here, we propose controlling the hydrogen bond network of interfacial water using an external electric field, which may induce structural changes or phase transitions. However, because the interfacial water layer is confined in an extremely thin space, it is difficult to directly observe its structural and phase transitions using conventional experimental methods. Therefore, a comprehensive understanding of the phase behavior of interfacial water under an electric field remains a significant challenge in condensed matter physics.

Field-effect transistor (FET) devices using two-dimensional materials (2DMs), such as graphene or transition-metal dichalcogenides (TMDs), have been employed not only to investigate fundamental electronic properties^8–10^ but also to realize high-speed,^11–13^ functional,^14–16^ and sensing devices.^17,18^ Graphene is highly sensitive to local electric fields; for instance, changes in electrostatic potential induced by a single elementary charge are detectable.^19^ Therefore, graphene serves as an ideal probe for investigating the structural and phase transitions of interfacial water under an electric field.

So far, water molecules have been excluded from 2DM devices because they are believed to degrade transport characteristics. Adsorbed water can interact with the 2DM, which causes hysteretic behavior in the transfer curve,^20–23^ hole accumulation,^21,24^ reduced carrier mobility,^21,22^ and the creation of trap states.^25–27^ Although these phenomena are often attributed to the EDMs of water molecules, the interaction between EDMs and 2DMs has not been fully clarified. This is because the interactions of an EDM with the charge carriers are complicated by electrostatic potentials arising from other EDMs, charged impurities, the dielectric substrate, and external gate voltages. For instance, it is well known that water molecules can induce hole doping in graphene.^21,24^ However, this doping mechanism cannot be fully understood only by considering EDMs. Another example is the location-dependent effect of water molecules. Water or ice deposited on the top surface of graphene produces no remarkable effect,^24,28^ whereas water molecules located underneath the graphene have a negative impact, as described above.^20–23^ These examples suggest complex interactions of water molecules placed between the substrate and the channel material. This implies that a more comprehensive mechanism must be considered to explain the effects induced by water molecules.

Here, we study the relationship between the configuration of EDMs and transport properties by preparing graphene FETs (GFETs) on hydrophilic SiO_2_/Si substrates. The density and scattering rate of charge carriers in graphene are highly sensitive to the surrounding environment. By investigating transport properties under various gate voltage *V*_g_ and temperature *T*, we clarify the interactions of a water molecule with other molecules, charged impurities, SiO_2_, and graphene, and examine their controllability via *V*_g_ and *T*.

## Results and Discussion

### Sample Preparation

Hydrophilic treatment of the SiO_2_/doped Si substrate was performed using oxygen plasma etching. This process generates silanol groups on the SiO_2_ surface, facilitating the adsorption of water molecules.^22^ The hydrophilicity of the substrate was confirmed by measuring the water contact angle, which decreased significantly from 51 ± 1° to 10 ± 1° after oxygen plasma etching, as shown in Figure S1 in the Supporting Information. GFETs were fabricated on these substrates using conventional microfabrication techniques. Figure 1(a,b) show the schematic side view and optical microscope image of the prepared GFET, respectively.

**Figure 1:**
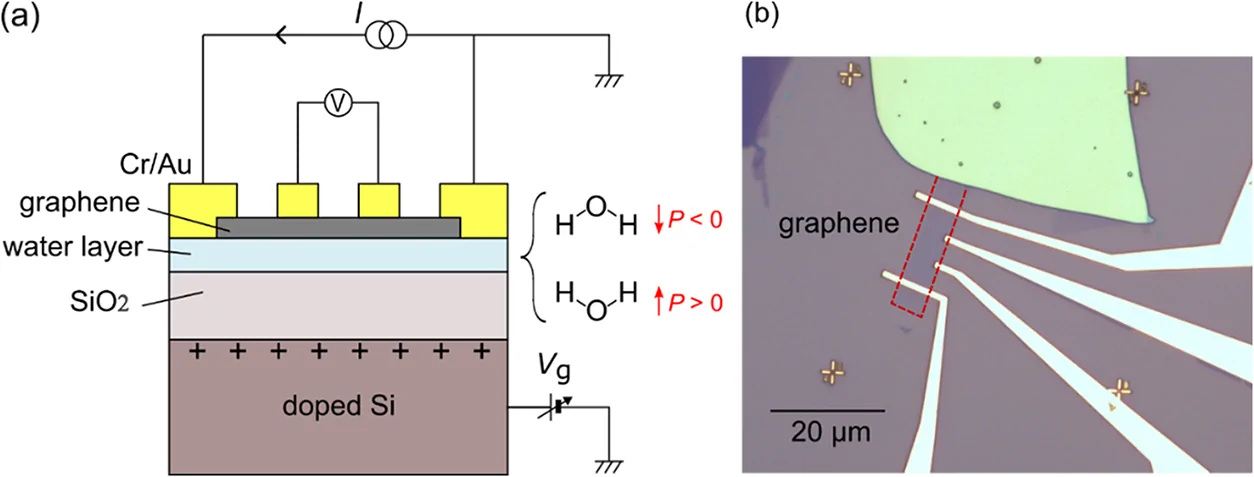
(a) Schematic side view of a GFET prepared on a hydrophilic substrate. The polarization directions of water molecules are indicated. (b) Optical microscope image of the prepared device.

### Temperature Dependence of Conductivity–Gate Voltage Curves

The sheet conductivity, σ, was measured by sweeping *V*_g_ between 0 and 40 V with decreasing *T*. First, σ was recorded from 298 K down to 88 K in steps of 20 K, called “Run 1” (Figure 2(a)). Next, σ was recorded from 268 to 188 K in steps of 5 K, called “Run 2” (Figure 2(b)). When *T* was decreased, the *V*_g_ was kept at 0 V, a procedure termed “zero electric field cooling (ZEFC) mode”. The measurement method is schematically illustrated in Figure S2­(a) in the Supporting Information. The dependence of the σ on *V*_g_ at a fixed temperature is defined as a σ–*V*_g_ curve. The raw σ–*V*_g_ curves are shown in Figure S3 in the Supporting Information. As shown in Figure S3, σ at *V*_g_ = 0 V slightly increases as *T* decreases. For clarity, σ–*V*_g_ curves are plotted in Figure 2(a,b) with offsets of −0.3 and −0.2 mS for Run 1 and Run 2, respectively.

**Figure 2:**
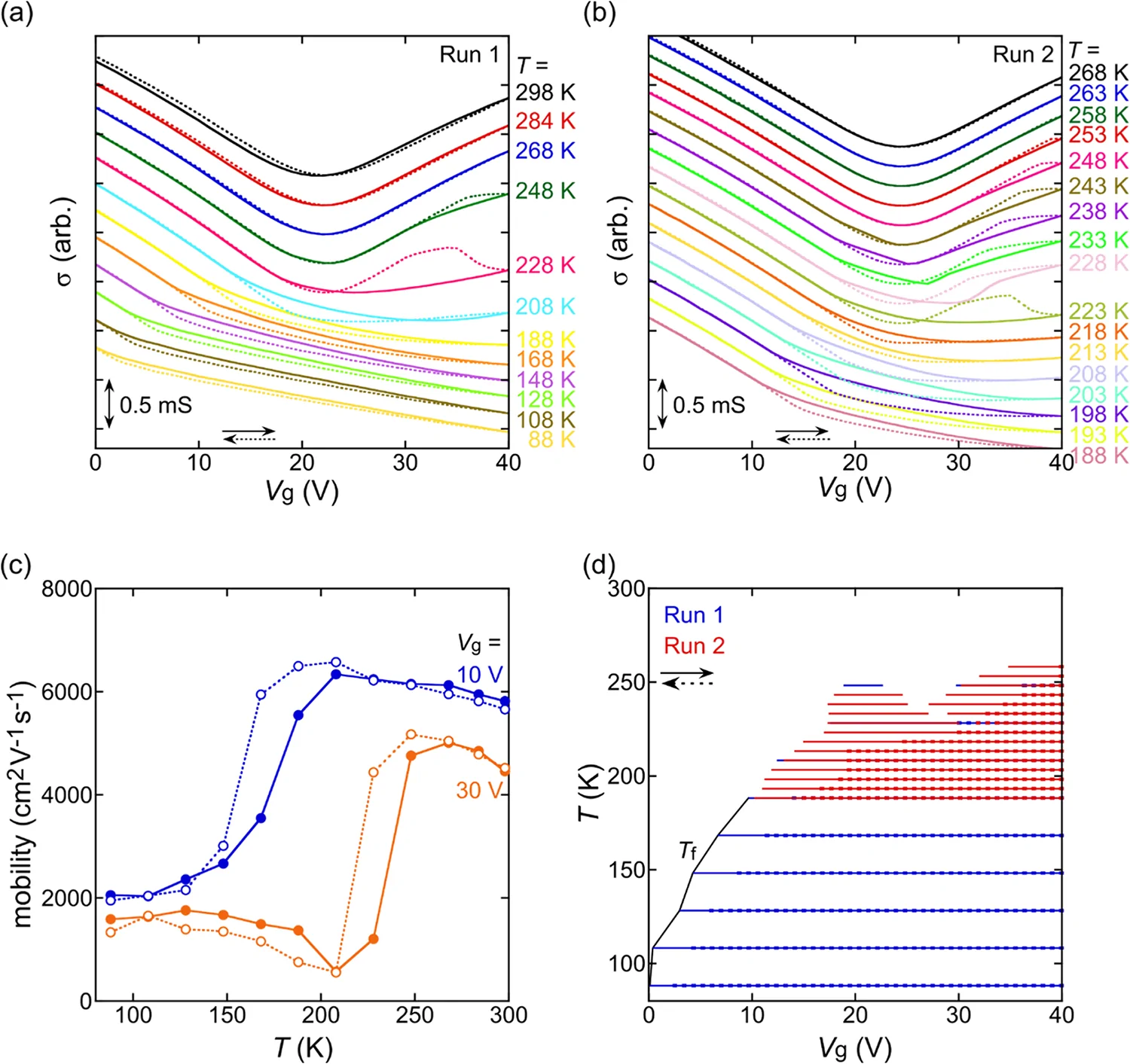
(a, b) σ–*V*_g_ curves measured with decreasing temperature. The curves are offset with −0.3 and −0.2 mS in (a) and (b), respectively. (c) Temperature dependence of field-effect mobility at *V*_g_ = 10 and 30 V evaluated from (a). (d) Regions where water molecules do not change their orientation freely in response to *V*_g_ are indicated with solid and dashed lines for forward and backward sweeps, respectively. The regions determined from (a, b) are shown in blue and red, respectively. The black line connecting the edge of the solid lines indicates the freezing point, *T*_f_.

The σ–*V*_g_ curves are classified into three stages at different temperature ranges. At 298–258 K (Stage I), a typical V-shaped σ–*V*_g_ curve is observed, although the charge neutrality point *V*_n_ is located at *V*_g_ = 22.0 V, indicating significant hole doping of the graphene. The field-effect mobility μ_FE_ is defined as μ_FE_ = (1/*C*_SiO_2__)|dσ/d*V*_g_|, where *C*_SiO_2__ = 11.5 nF cm^–2^ is the capacitance of the SiO_2_ layer. We evaluate the electron and hole mobilities, μ_FE_^e^ and μ_FE_^h^, to be 4.5 × 10^3^ and 5.6 × 10^3^ cm^2^V^–1^s^–1^, respectively, in the electron and hole regimes of the σ–*V*_g_ curve (see Figure 2a). These values are comparable to those generally obtained for GFET devices prepared on the hydrophobic substrates.^22^ All 11 devices prepared on the hydrophilic substrate showed consistent results. The average charge neutrality point was ⟨*V*_n_⟩ =15.1 ± 7.7 V, and the average mobilities were ⟨μ_FE_^e^⟩ = (6.2 ± 2.8) × 10^3^ cm^2^V^–1^s^–1^ and ⟨μ_FE_^h^⟩ = (7.6 ± 3.3) × 10^3^ cm^2^V^–1^s^–1^ at room temperature (RT). Unlike previous reports,^21,22^ we observed neither significant hysteresis nor a reduction in μ_FE_^e^ and μ_FE_^h^. Hysteresis in the σ–*V*_g_ curve is ascribed to the reversal of EDMs while sweeping *V*_g_.^23^ The polarity of a dipole layer and its inversion by *V*_g_ can be induced by the interaction of an EDM with the charged substrate or other EDMs. The absence of hysteresis in our results suggests that the interactions between EDMs are weaker than the thermal energy, allowing each EDM to change its orientation independently and promptly in response to the *V*_g_ in our system.

However, as the temperatures decreased, the σ–*V*_g_ curves exhibited hysteretic behavior in the range of 253–208 K (Stage II). This indicates that the interaction between EDMs became comparable to or stronger than the thermal energy. Consequently, the orientation of the EDMs failed to keep pace with the sweep of *V*_g_. In other words, the response of the EDMs to changes in *V*_g_ was delayed due to these interactions, resulting in hysteresis.

With further temperature decrease, the hysteretic behavior was suppressed, and the slope of the σ–*V*_g_ curve significantly decreased in the high *V*_g_ region below 188 K (Stage III). The suppression of hysteresis suggests a fixed orientation of the EDMs, indicating a phase transition from the liquid to the solid state of water molecules. The slope of the σ–*V*_g_ curve decreased upon transitioning from the liquid state to the solid state. Thus, the inflection point in the σ–*V*_g_ curve defines the freezing point *T*_f_. The value of *T*_f_ was 88 K at *V*_g_ = 0 V, which is significantly lower than the 273.15 K freezing point of bulk water. This observation can be understood as follows: water molecules form a thin layer or small clusters on hydrophilic SiO_2_, with an average thickness of 2-3 monolayers.^29–36^ Such thin water layers freeze into amorphous ice^37^ rather than crystalline ice. Admittedly, the glass transition temperature of low-density amorphous ice was measured to be 136 K.^38^ According to molecular dynamics (MD) simulations, water confined in a narrow space can exhibit a much lower *T*_f_.^39^ Furthermore, MD simulations of water layers confined in narrow spaces have demonstrated electric-field-induced phase transition.^40,41^ Since the water molecules in our system are confined in a restricted region with charged boundaries, *T*_f_ likely depends on *V*_g_.

Since the device was cooled in the ZEFC mode, the EDMs at *V*_g_ = 0 V froze into the same configuration in all Stages I, II, and III. However, the slopes of σ–*V*_g_ curves in Stage I and III are drastically different. The temperature dependence of μ_FE_, evaluated at *V*_g_ = 10 and 30 V, is shown in Figure 2(c). A significant reduction in μ_FE_ accompanies the transition from the liquid state (Stage I) to the ice state (Stage III). It is important to note that μ_FE_ is evaluated by assuming that the carrier density in graphene is modulated by *C*_SiO_2__ *V*_g_/*e*. If the ice layer acts solely as an additional capacitor that does not contribute to carrier accumulation, the μ_FE_ value of ∼2000 cm^2^V^–1^s^–1^ at low temperatures may represent the intrinsic mobility for GFETs on the hydrophilic SiO_2_. Here, the intrinsic mobility is defined as μ*_i_* = *el*/ℏ*k*_F_, where *l* and *k*_F_ are the mean free path and Fermi wave number, respectively.^42^ In contrast, the strong *V*_g_ dependence of σ in Stage I suggests that the free rotation of the EDMs contributes to the carrier accumulation, thereby yielding an apparent high mobility. Thus, the σ in Stage I is primarily modulated by the water molecules depending on their orientations.

Figure 2(d) schematically shows the region in the *V*_g_–*T* plane where water molecules cannot rotate freely. In other words, the regions where the slope of the σ–*V*_g_ curve deviates from that in the liquid phase are indicated by lines. The boundaries of these regions represent the phase transition between the liquid and non-liquid states (solid or an intermediate state). As shown in Figure 2(d), the interaction between EDMs is first observed at *V*_g_ ≥ 35 V at 258 K, and this region expands toward lower *V*_g_ as the temperature decreases. That is, a positive correlation between *T*_f_ and *V*_g_ is observed. More detailed analysis of the hysteretic behavior in the σ–*V*_g_ curves also indicate the same trend, as shown in Figure S4­(c) in the Supporting Information. Nevertheless, it is important to note that the phase boundary becomes complex around 240 K, where interactions between EDMs are simultaneously observed in both the electron and hole regimes (Figure 2(b)).

A careful examination of Figure 2(a) reveals slight hysteresis in the σ–*V*_g_ curve at 298 K. The initial and final values of σ measured at *V*_g_ = 0 V do not agree, indicating a slight increase in hole density after the *V*_g_ sweep. In fact, the value of *V*_n_ at RT shifted from 22.0 V at the beginning of Run 1, to 22.3 V at the end of Run 1, and finally to 23.2 V at the end of Run 2. This gradual shift in *V*_n_ indicates unintentional hole doping of the graphene during the measurements. The origin of the doping may be electron transfer from the graphene to the SiO_2_ substrate, as discussed later.

To verify the reproducibility of the results, several devices (device #1, #2, #3) on the hydrophilic SiO_2_ were measured with decreasing *T* under the same conditions as the above-mentioned device (device #0). The results were summarized in S8 in the Supporting Information. Overall features were reproduced as shown in Figure S8.

To further verify the role of interfacial water, a control experiment was performed using HMDS-treated hydrophobic substrates (see Sections S1 and S5 in the Supporting Information). For the GFET prepared on the hydrophobic SiO_2_, the transport properties exhibited little temperature dependence. The σ–*V*_g_ curves showed neither hysteretic behavior nor significant *T* dependence. The *V*_n_ was independent of *T*, and μ_FE_ slightly increased with decreasing *T*. This control experiment confirmed that the characteristic temperature dependences of σ–*V*_g_ curves, *V*_n_, and μ_FE_ observed in GFETs on hydrophilic SiO_2_ are attributed to the presence of confined interfacial water. These results support the interpretation that the behavior arises from the polarization and phase transitions of the interfacial water, as described previously.

### Temperature Dependence of Conductivity at Fixed Gate Voltage Measured in Different Modes

To clarify the interaction between EDMs, we measured the σ using two distinct methods, as illustrated in Figure S2 (b,c) in the Supporting Information. One is the electric field cooling (EFC) mode. In this mode, *V*_g_ is applied at RT, and the device is cooled to the lowest temperature, *T*_min_ (=84 K). The σ is then successively measured while increasing the temperature under a constant *V*_g_. The other is the zero electric field cooling (ZEFC) mode, as described previously. In this mode, the device is cooled to *T*_min_ at *V*_g_ = 0 V, after which *V*_g_ is applied. The σ is measured while increasing temperature. In the EFC mode, the EDMs freeze into an equilibrium state corresponding to the applied *V*_g_, whereas in the ZEFC mode, they freeze into a non-equilibrium state. During the heating process, the phase transition from the non-equilibrium solid state to the equilibrium liquid state can be precisely detected from the inflection points in the σ–*T* plots. This approach is analogous to the comparison of magnetization measured under field cooling (FC) and zero-field cooling (ZFC) conditions, which has been widely used to study interactions between magnetic moments.^43–45^ Similarly, we applied this method in the present study to identify the electric polarization, *P*, of water molecules, *i.e.*, the interaction between EDMs.

Figure 3 shows the temperature dependence of the σ measured in the EFC mode (dashed lines) and the ZEFC mode (solid lines) at various gate voltages. In all measurements, the temperature was raised from *T*_min_ to RT over a period of 1 h with a heating rate of 3.5 K min^–1^. In the EFC mode, σ changes smoothly with temperature because the EDMs remain in an equilibrium state at each *V*_g_. In the ZEFC mode, on the other hand, σ­(*T*) exhibits abrupt changes at several temperatures, which are indicated by open/closed circles. Note that the change in σ­(*T*) under a constant *V*_g_ indicates that *P* of the water layer reflects the carrier density in graphene.

**Figure 3:**
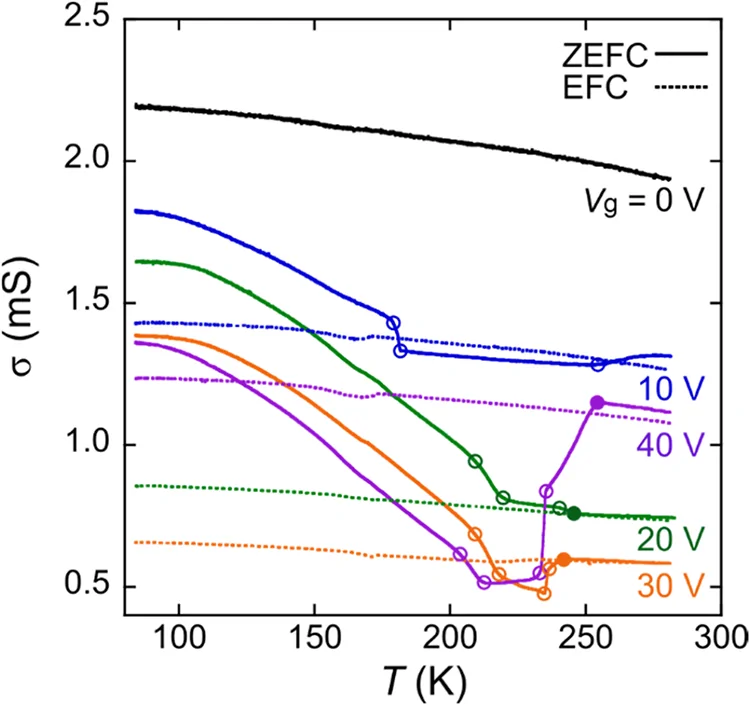
Conductivity measured in the EFC and ZEFC modes, indicated by dashed and solid lines, respectively. Several turning points observed in the ZEFC mode are indicated by circles. Among them, the melting point is indicated by closed circles. These points define the boundaries between the relaxing solid state, the stable solid state, and the liquid state.

Based on these observations, the states of water molecules are classified into four distinct states. Taking the case of *V*_g_ = 40 V as an example, the states are distinguished by temperature as follows:

(i) At temperature near *T*_min_ (∼84 K), the slope of σ­(*T*) agrees with that observed in the EFC mode. In this temperature regime, the EDMs are completely frozen with a negative *P*, which accumulates holes in the graphene as shown in Figure 2(a). This is defined as the “frozen solid state”, a non-equilibrium state characterized by a constant negative *P*.
(ii) As the temperature increases, σ gradually decreases above 100 K. This regime is termed the “relaxing solid state” in which negative *P* gradually increases, indicating gradual relaxation of the EDMs toward a stable state. The boundary between the frozen solid state and the relaxing solid state near *T*_min_ is not plotted in Figure 3, as it could not be clearly identified; it will be determined by ZEFC measurements at lower temperatures.
(iii) At 204 K, σ abruptly decreases and remains almost constant between 213 and 233 K. Notably, σ reaches its minimum value in this region, indicating that the graphene is charge-neutral. This implies zero net polarization or a random orientation of the EDMs, assuming that the carrier density is primarily determined by *P* and not significantly affected by *V*_g_ in this regime. This regime is referred to as the “relaxed solid state” or “stable solid state” characterized by the absence of net polarization (*P* ∼ 0).
(iv) With further temperature increase, σ abruptly rises and eventually agrees with the EFC data at *V*_g_ = 40 V. The temperature at which the σ of the ZEFC mode converges with that of the EFC mode is defined as the melting temperature, *T*_m_, indicated by closed circles in Figure 3. The state above *T*_m_ (254 K for this case) is an equilibrium “liquid state” with a positive *P*.

The σ­(*T*) curves for other gate voltages are classified in a similar manner. The EDMs transition from the relaxing solid state to the liquid state via the stable solid state (relaxing solid → stable solid → liquid). For *V*_g_ = 30 and 40 V, the carrier type in graphene changes from holes at *T*_min_ to electrons at RT, passing through the charge neutrality point in the stable state. Despite the different σ values at RT for 30 and 40 V, the σ values are almost identical in the stable solid state region (220–230 K). This indicates that this state is determined only by the interactions between EDMs and is unaffected by *V*_g_. For *V*_g_ = 10 and 20 V, the carriers remain holes throughout the entire temperature range; nevertheless, the stable state with a constant negative *P* is clearly observed. The *T*_m_ at *V*_g_ = 10 V is not plotted due to its ambiguity. The σ at *V*_g_ = 20 V slightly decreased above *T*_m_, which differs from the behavior observed at other *V*_g_s. This suggests that the stable solid state in the hole regimes is inhomogeneous, comprising both relaxed (zero polarization) and unrelaxed (negative polarization) water molecules. The distinct behavior at the melting point can be attributed to the different ratio of these states as explained in S9 in the Supporting Information.

To demonstrate the reliability of these results, additional measurements were performed with different heating rates and repeated cooling and heating cycles (Figures S6 and S7). Slower heating rates cause the σ­(*T*) curves to shift slightly toward lower temperatures, likely reflecting the kinetics of the relaxing solid state. This slower heating rate facilitates the rearrangement of the hydrogen bond network, allowing confined water molecules to reach an equilibrium relaxed solid state at lower temperatures. However, the overall σ­(*T*) profiles remain consistent, indicating an identical phase transition mechanism. Furthermore, repeated measurements show nearly identical transition temperatures. These findings confirm that the inflection points in the σ­(*T*) curves are highly reproducible and intrinsic to the phase transition of confined water.

### Phase Diagram of the Water and Ice Layers on Hydrophilic SiO_2_

Figure 4 shows the phase diagram in the *V*_g_–*T* plane, constructed from Figures 2(d) and 3. The non-liquid regions identified from the forward and backward sweeps in Figure 2(d) are enclosed by solid and dashed lines, respectively. The characteristic temperatures determined from the inflection points in Figure 3 are plotted as circles, with the melting point *T*_m_ indicated by closed circles. The stable solid state is shaded in green. The boundary in Figure 2(d), which separates the regions of free and restricted EDM rotation, agrees well with *T*_m_ at each *V*_g_, except for *V*_g_ = 10 V. At *V*_g_ = 10 V, the *T*_m_ > 255 K observed in the ZEFC mode lies within the liquid state region of the σ–*V*_g_ curve. This discrepancy may be attributed to the superheating of the stable solid state in the ZEFC mode. In addition to distinguishing between the liquid and non-liquid states, the ZEFC measurements provide more detailed insights into the relaxing solid, stable solid, and liquid states. Furthermore, a detailed examination of the ZEFC data reveals the presence of a transition state between the stable solid and liquid states. This transition state and the stable solid state correspond to the temperature range of Stage II in Figure 2(a,b). It is inferred that the strong interactions between EDMs give rise to the complex hysteretic behavior observed in the σ–*V*_g_ measurement, as well as the stable polarization state in the ZEFC measurement.

**Figure 4:**
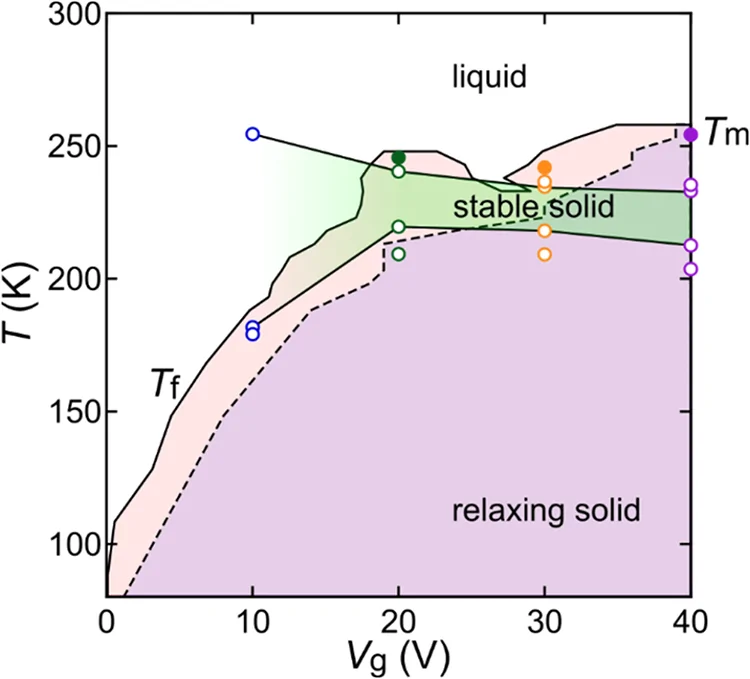
Phase diagram of water molecules in the gate voltage–temperature plane. The regions derived from Figure 2(d) are enclosed by solid and dashed lines. The inflection points identified in Figure 3 are plotted as circles, with *T*_m_ indicated by closed circles. The stable solid state is shaded in green.

The *T*–*V*_g_ phase diagram presented here is based on a phase transition mechanism fundamentally different from that of the conventional temperature-pressure (*T*–*p*) phase diagram.^46–48^ In the latter case, phase transitions are driven by hydrostatic pressure, which alters the free-energy by changing the intermolecular distance. In contrast, the *T*–*V*_g_ phase diagram reported here is governed by an electric field that couples with the EDMs of water molecules, thereby modifying their molecular orientation and hydrogen bond network. Our results demonstrate that the electric field serves as an effective tuning parameter for controlling the phase behavior of nanoconfined water. Since reversible and continuous gate voltage control can be easily integrated with electronic devices, this approach offers new opportunities not only for studying the fundamental properties of confined water by manipulating the hydrogen bond network but also for developing novel electronic devices that utilize the EDMs of water molecules.

### Discussion on the Electric Polarization and Phase Transition of Water Molecules

Here, we discuss the phase transition of water molecules in relation to their polarization. First, we consider the polarization of water molecules in the *V*_g_–*T* plane. The configuration of water molecules on hydrophilic SiO_2_ has recently been investigated using heterodyne-detected vibrational sum-frequency generation (VSFG) spectroscopy.^29^ It was reported that water molecules orient their hydrogen atoms toward the air at low relative humidity; this orientation was attributed to the strong hydrogen bond between the oxygen atom of the water molecule and the hydrogen atom of the silanol group. This upward (positive) polarization contrasts with our results, which show downward (negative) polarization at *V*_g_ = 0 V and RT. The difference between their experiments and ours lies in whether the water molecules are exposed to the air or confined under the graphene. Therefore, the downward polarization may be ascribed to the presence of the graphene layer. Considering the hole doping of graphene, electron transfer from the graphene to the SiO_2_ substrate is a plausible explanation for this negative polarization. The destination of these electrons can be understood by considering defects on the amorphous SiO_2_ surface. Silicon or oxygen atoms that are not fully coordinated^49,50^ possess dangling bonds. An electron can be transferred from the graphene to the dangling bond of a defect, forming a negatively charged lone pair. As a result, the negatively charged lone pair orients the water molecules downward, thereby inducing hole doping in the graphene. Furthermore, the unintentional hole doping observed during the σ­(*V*_g_) measurements is also explained by this electron transfer mechanism, as verified by the shift of *V*_n_.

The carrier density in graphene, controlled by *V*_g_ at RT, is schematically illustrated in Figure 5(a). Based on the ZEFC measurement results, the carrier density is significantly influenced by *P*. At *V*_g_ = 0 V, the negative *P*, caused by negatively charged lone pairs, accumulates holes in graphene. As *V*_g_ increases, the electric field, *E*, generated by *V*_g_ and the negative charge cancel each other out at *V*_g_ = *V*_n_, rendering the graphene charge-neutral. A further increase in *V*_g_ reverses the sign of *P* to positive, which in turn accumulates electrons in the graphene.

**Figure 5:**
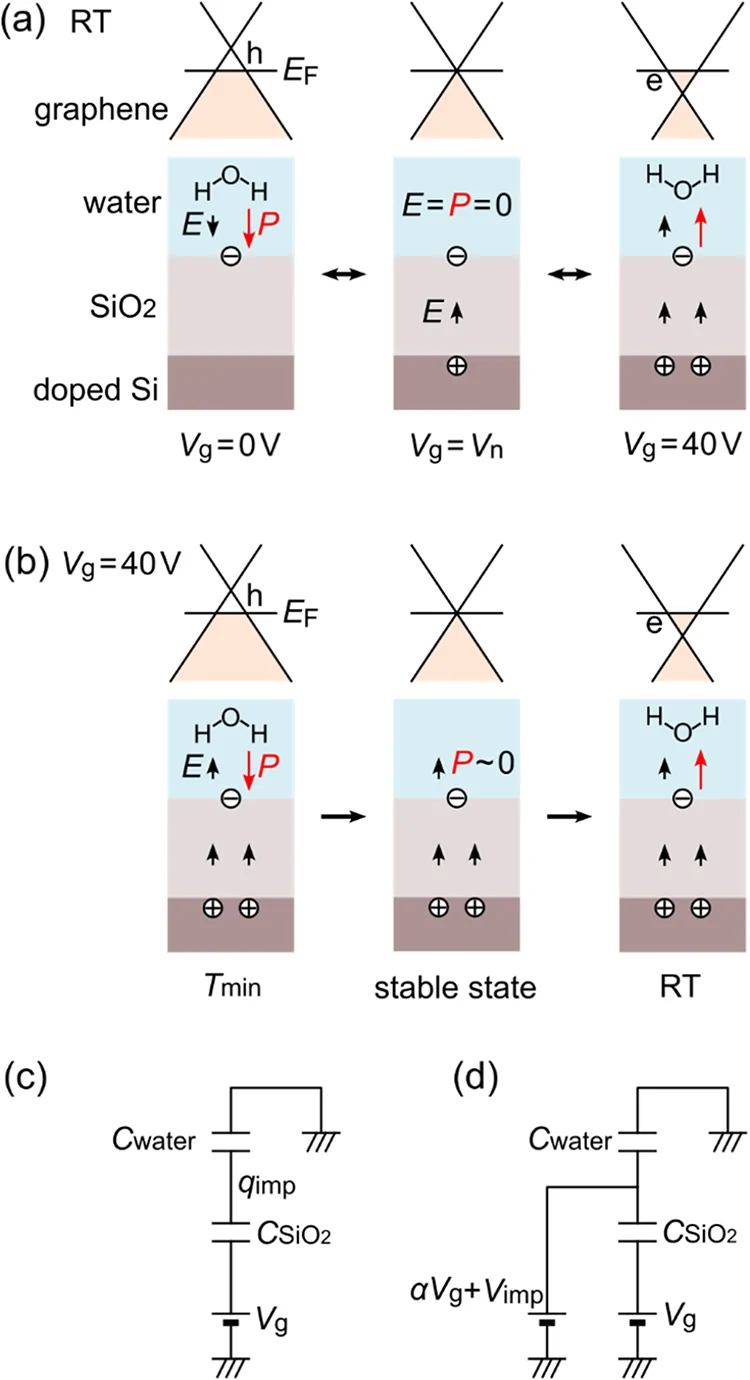
(a) Schematic diagram of the *V*_g_ dependence of the graphene carrier density, controlled by the electric polarization *P*. (b) Schematic diagram of the *T* dependence of the graphene carrier density in the ZEFC mode at *V*_g_ = 40 V. (c) Conventional two capacitors model, which fails to explain carrier accumulation due to water molecule polarization. The impurity charge is denoted by *q*_imp_. (d) Model for the water layer accumulating charge carriers independently of *V*_g_. The parameters α ≳ 0 and *V*_imp_ represent the effects of *P* on carrier accumulation and the impurity charge, respectively.

Figure 5(b) shows the evolution of the carrier density in the ZEFC mode for *V*_g_ = 40 V. The interaction between EDMs gradually weakens with increasing temperature. The negative *P* frozen at *T*_min_ gradually increases until it becomes zero in the stable solid state. A further temperature increase causes *P* to become positive.

Next, we discuss the relationship between the polarization and the phase diagram. We observed an extremely low *T*_f_ (∼88 K) at *V*_g_ = 0 V, along with a positive correlation between *T*_f_ and *V*_g_. One possible reason for such a low *T*_f_ at *V*_g_ = 0 V, where the polarization is negative, is that the freezing of electrically polarized water is unstable. This is because the orientations of hydrogen bonds in crystalline ice are random to minimize the total electric polarization. In this scenario, if *T*_f_ is determined by the magnitude of *P*, the phase diagram would be expected to be symmetric with respect to *V*_n_. That is, *T*_f_ should not increase monotonically with *V*_g_; rather, it should reach a maximum at *V*_n_ (where *P* = 0) and decrease for *V*_g_ > *V*_n_ where *P* is positive. Indeed, the signature of this symmetric phase transition is observed in the hysteretic behaviors in both electron and hole regimes around *T* = 240 K, as well as in the stable state observed in the ZEFC mode for each *V*_g_. To further establish the correlation between *T*_f_ and *P*, measurements at much larger *V*_g_ are required.

Finally, we discuss the reason why the slopes of the σ–*V*_g_ curves in Figure 2(a,b), or the μ_FE_ in Figure 2(c) differ between the water (Stage I) and ice (Stage III) states. As shown in the σ–*T* curve in the ZEFC mode at *V*_g_ = 40 V (Figure 3), the carrier type changes from holes to electrons solely due to the reversal of the polarization. This situation differs from that of a conventional parallel-plate capacitor with a dielectric, in which *P* is determined by *E* produced by the free charges on the electrodes. In contrast, in our system, the carrier density in graphene is directly affected by the *P* of the water/ice layer. This is likely because the low carrier density and low density of states (DOS) of graphene make it highly susceptible to environmental charges. Consequently, our device cannot be described by the conventional series connection of two capacitors—the water/ice layer and the SiO_2_ layer—as illustrated in Figure 5(c). In such a conventional model, the gate voltage dependence of the carrier density is determined by the total capacitance, *C*_total_ = *C*_water_ *C*_SiO_2__/(*C*_water_ + *C*_SiO_2__) or *C*_ice_ *C*_SiO_2__/(*C*_ice_ + *C*_SiO_2__). Even if *C*_water_ and *C*_ice_ are much larger than *C*_SiO_2__, the total capacitance *C*_total_ is approximately equal to *C*_SiO_2__ and remains almost identical for both the water and ice layers.

To account for the fact that the polarization induces carriers, the two capacitors must operate distinctly, as shown in Figure 5(d). Here, the carrier density in graphene is primarily determined by the polarization of the water/ice layer, which is modified by *V*_g_. The voltage across *C*_water_ should be larger than that across *C*_ice_, or the parameter *α* indicated in Figure 5(d) should be larger in the water state than in the ice state. This explains the enhanced *V*_g_ dependence of σ in the water state. The precise carrier density and carrier mobility can be experimentally evaluated using Hall effect measurements. Such experiments are currently in progress.

## Conclusions

We investigated the effects of water molecules adsorbed on hydrophilic substrates on the transport properties of graphene. Contrary to previous reports, the carrier mobility of GFETs is not degraded compared to that of GFETs prepared on hydrophobic substrates. The transfer characteristics changed significantly with decreasing temperature, indicating a phase transition from water to ice that depends on the gate voltage. At RT, the free movement of water molecules affects the carrier density, thereby enhancing the apparent field-effect mobility. This phenomenon demonstrates the potential utility of water molecules, in contrast to the commonly accepted notion that water molecules merely degrade transport characteristics. Furthermore, since the effect of water molecules on the carrier density of graphene cannot be explained by the conventional capacitor model, the fundamental principles of capacitors should be reexamined from a nanoscopic viewpoint. In conclusion, interfacial water should not always be regarded as an undesirable contaminant in 2DMs. Under controllable conditions, its polarization can enhance electrostatic gating. This finding provides a new perspective on the role of interfacial water in 2DM-based electronic devices.

We also presented a phase diagram of liquid water and amorphous ice states confined between graphene and a hydrophilic SiO_2_. While phase diagrams have typically been constructed in temperature-pressure planes, our results show that an electric field can be introduced as a new controllable parameter. Our GFET-based method can be applied not only to study the novel structure of amorphous ice but also to investigate the polarization of dielectric materials.

Although the present work provides new insights into the phase behavior of confined interfacial water, we note several limitations of our study. First, the proposed phase transition is inferred from the electrostatic response of graphene measured via transport measurements, rather than from direct structural characterization of the confined water layer using techniques such as neutron diffraction^51,52^ or VSFG spectroscopy.^29,53^ Previous theoretical studies^54–56^ have shown that confined water often forms amorphous ice under strong nanoconfinement and at low temperatures. These theoretical results support our inference that the confined water freezes into an amorphous structure, likely due to the amorphous nature of the SiO_2_ boundary. Second, the thickness of the interfacial water was not systematically controlled. Future studies combining humidity-controlled fabrication of GFETs with advanced structural characterization techniques and *in situ* transport measurements at cryogenic temperatures will enable a more quantitative understanding of the electric-field-induced phase transition mechanism. Specifically, such studies will clarify the relationship between interfacial water thickness and polarization-induced phase transition.

## Methods

GFETs were fabricated in a cleanroom maintained at a temperature of 24°C and relative humidity of 26%. Si substrates with a 300 nm thermally oxidized SiO_2_ layer were cleaned using organic solvents and ultrapure water. Hydrophilic treatment of the substrates was performed via oxygen plasma etching (250 W for 30 s) using a plasma reactor (PR41, Yamato Scientific Co., Ltd.). The hydrophilicity was confirmed by measuring the water contact angle using a contact angle meter (ECA-1, Kyowa Interface Science Co., Ltd.). Graphene flakes were transferred onto the substrates from bulk graphite with micromechanical cleavage within 2 h after oxygen plasma treatment. Monolayer graphene devices were prepared using conventional microfabrication techniques, including electron-beam lithography and metal deposition. Electrical measurements were performed in vacuum less than 10^–2^ Pa in a cryostat (MicrostatHe, Oxford Instruments) with flowing liquid nitrogen. The temperature was regulated using a temperature controller (ITC 503S, Oxford Instruments), and the sample temperature was monitored at the sample stage. The minimum temperature reached was 84 K. The cooling rate to measure σ–*V*_g_ curve is 1.0 K min^–1^, and that for EFC and ZEFC modes is 3.5 K min^–1^. Four-terminal conductivity measurements were conducted using a Keithley system source meter (models 2635A and 2602A).

## Supplementary Material



## Notes

Contact angle measurement to assess hydrophilicity; schematic representation of measurement methods; σ–*V*_g_ curves with decreasing temperature; analysis of hysteretic behaviors in σ–*V*_g_ curves; transport properties of GFETs prepared on hydrophobic substrates; reproducibility of ZEFC measurements over multiple cooling–heating cycles; reproducibility of ZEFC measurements for different heating rates; reproducibility of σ–*V*_g_ curve for plural devices; conductivity at the stable solid state at *V*_g_ = 10 and 20 V (PDF)
