# Supplementary material for: Interactions of Water Molecules Adsorbed on Hydrophilic Substrates Clarified by Transport Characteristics of Graphene
Source: ACS Omega. 2026 Sep 8;11(37):56014–22. doi: 10.1021/acsomega.6c05969 (PMC13613453; doi:10.1021/acsomega.6c05969)
Supplement: Supplementary file 1 [file ao6c05969_si_001.pdf]

Supporting Information

Interactions of Water Molecules Adsorbed on Hydrophilic Substrate Clarified by Transport Characteristics of Graphene

Yi Chen, Misuzu Kitahara, Lei Zhi, Yoshihiro Kubozono, Hidenori Goto*

Research Institute for Interdisciplinary Science, Okayama University, Okayama 700-8530, Japan

Contents

S1. Contact angle measurement to assess hydrophilicity

S2. Schematic representation of measurement methods

S3. $\sigma - V_g$ curves with decreasing temperature

S4. Analysis of hysteretic behaviors in $\sigma - V_g$ curves

S5. Transport properties of GFETs prepared on hydrophobic substrates

S6. Reproducibility of ZEFC measurements over multiple cooling–heating cycles

S7. Reproducibility of ZEFC measurements for different heating rates

S8. Reproducibility of $\sigma - V_g$ curve for plural devices

S9. Conductivity at the stable solid state at $V_g =$ 10 and 20 V

S1. Contact angle measurement to assess hydrophilicity

Hydrophilic treatment of substrates was performed with oxygen plasma etching, as described in the Methods section. For control experiment, hydrophobic treatment of substrates was also carried out using 1,1,1,3,3,3-Hexamethyldisilazane (HMDS) after oxygen plasma etching. The change in the hydrophilicity of the substrate was confirmed by measuring the water contact angles. The average contact angles and standard deviations, measured at more than 5 different points, were $51 \pm 1°$, $10 \pm 1°$, and $58 \pm 1°$ before and after oxygen plasma treatment and hydrophobic treatment with HMDS, respectively. The change in the contact angle is shown in Figure S1.

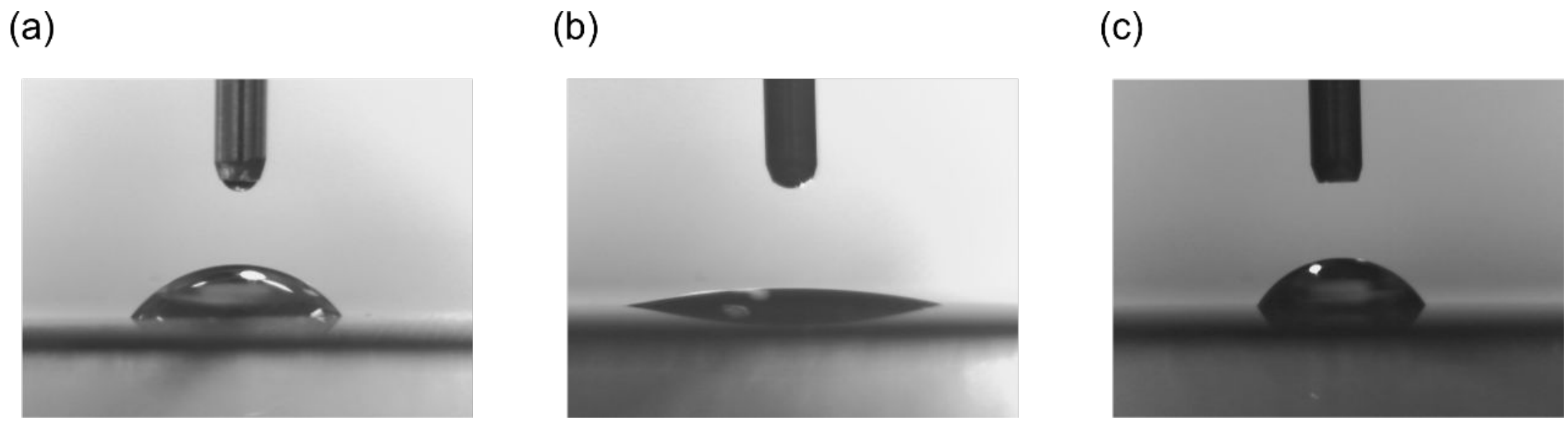

Figure S1. Contact angles of water droplets on SiO_2/Si substrates (a) before and (b) after oxygen plasma treatment, and (c) after HMDS treatment. The contact angle significantly decreased with oxygen plasma from 52.6° in (a) to 9.6° in (b), while it in turn increased to 59.3° with hydrophobic treatment in (c).

S2. Schematic representation of measurement methods

Figure S2 illustrates the measurement methods for determining the conductance in the $V_g - T$ plane in this study.

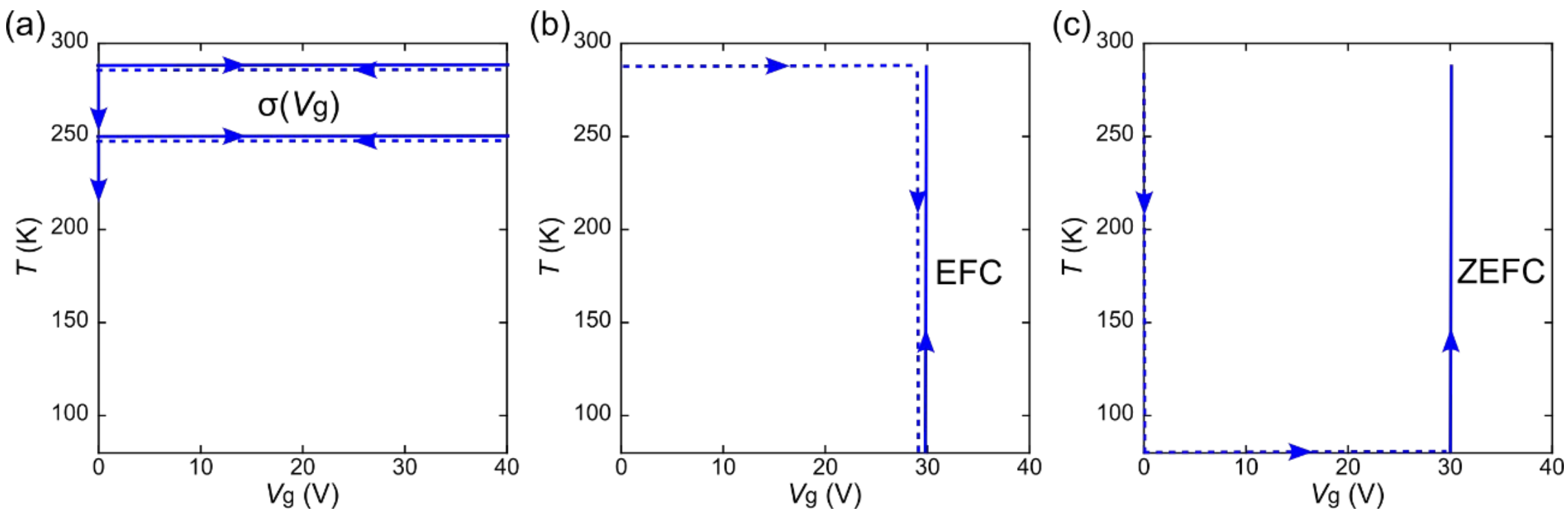

Figure S2. Schematic representation of the measurement methods. (a) $\sigma - V_g$ measurement. (b) Electric field cooling (EFC) measurement. (c) Zero electric field cooling (ZEFC) measurement.

S3. $\boldsymbol{\sigma - V_g}$ curves with decreasing temperature

The $\sigma - V_g$ curves were measured during cooling in Run 1 and Run 2, using different temperature steps. The raw data are shown in Figure S3. For clarity, the curves presented in Figure 2(a) and (b) of the main text are offset vertically.

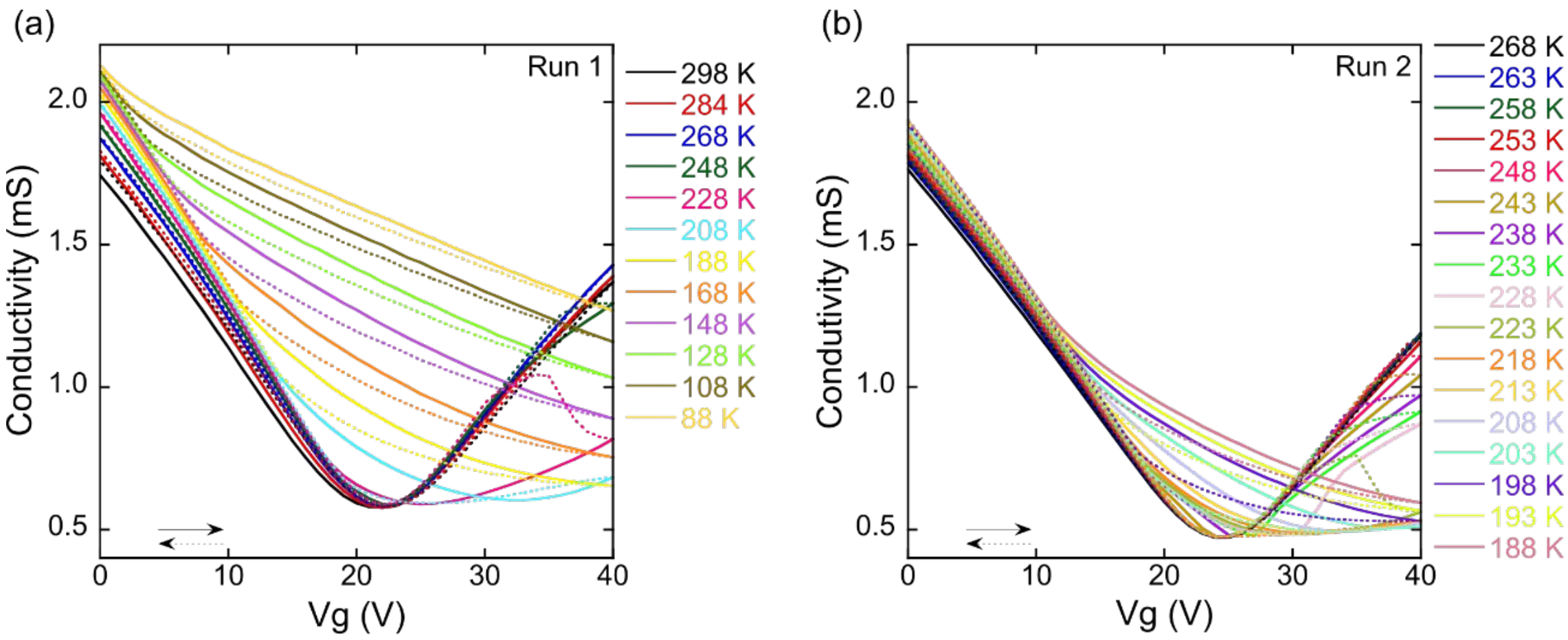

Figure S3. $\sigma - V_g$ curves measured during decreasing temperature. (a) Run 1 measured in 20 K steps. (b) Run 2 measured in 5 K steps.

S4. Analysis of hysteretic behaviors in $\boldsymbol{\sigma - V_{\mathrm{g}}}$ curves

As seen in Figure 2(a) and (b) or Figure S3(a) and (b), the charge neutrality point, V_{n} (defined as the gate voltage at which the conductivity is minimum), gradually increases and eventually exceeds the measurement range as the temperature decreases. The values of V_{n} are plotted in Figure S4(a). The filled and open symbols correspond to the upward and downward voltage sweeps, respectively. The discrepancy in V_{n} between the two sweeps arises from the hysteretic behavior of the $\sigma - V_{\mathrm{g}}$ curves, indicating that the interactions between molecules become significant.

To investigate these interactions, the hysteresis in the $\sigma - V_{\mathrm{g}}$ curves was analyzed in detail. The magnitude of hysteresis, S_{hys}, was estimated by integrating the area enclosed by the upward and downward $\sigma - V_{\mathrm{g}}$ curves, using

$$S_{\mathrm{hys}} = \int_{0}^{40} \{\sigma_{+}(V_{\mathrm{g}}) - \sigma_{-}(V_{\mathrm{g}})\} dV_{\mathrm{g}}\,, \qquad \text{(S1)}$$

where σ_{+} and σ_{-} represent the conductance during the forward and backward sweeps, respectively. This value, S_{hys}, is plotted in Figure S4(b). As the temperature decreases, S_{hys} exhibits a sharp dip followed by a peak. This trend suggests that the hysteretic behavior first emerges in the hole regime and then shifts to the electron regime at lower temperatures.

To estimate the gate voltage at which the hysteresis becomes significant, the weighted

average gate voltage, $V_{\mathrm{g,\,hys}}$, was calculated as:

$$V_{\mathrm{g,\,hys}} = \frac{\int_0^{40} V_{\mathrm{g}} \left| \sigma_+(V_{\mathrm{g}}) - \sigma_-(V_{\mathrm{g}}) \right| dV_{\mathrm{g}}}{\int_0^{40} \left| \sigma_+(V_{\mathrm{g}}) - \sigma_-(V_{\mathrm{g}}) \right| dV_{\mathrm{g}}} \tag{S2}$$

Here, $V_{\mathrm{g,\,hys}}$ represents the gate voltage averaged with weights proportional to the magnitude of the hysteresis. As shown in Figure S4(c), $V_{\mathrm{g,\,hys}}$ jumps suddenly at $T = 253$ K, and then gradually decreases with further cooling. This behavior is consistent with the analysis of S_{hys}, indicating that the interactions between EDMs initially occur in the hole regime and spread into the electron regime as the temperature decreases.

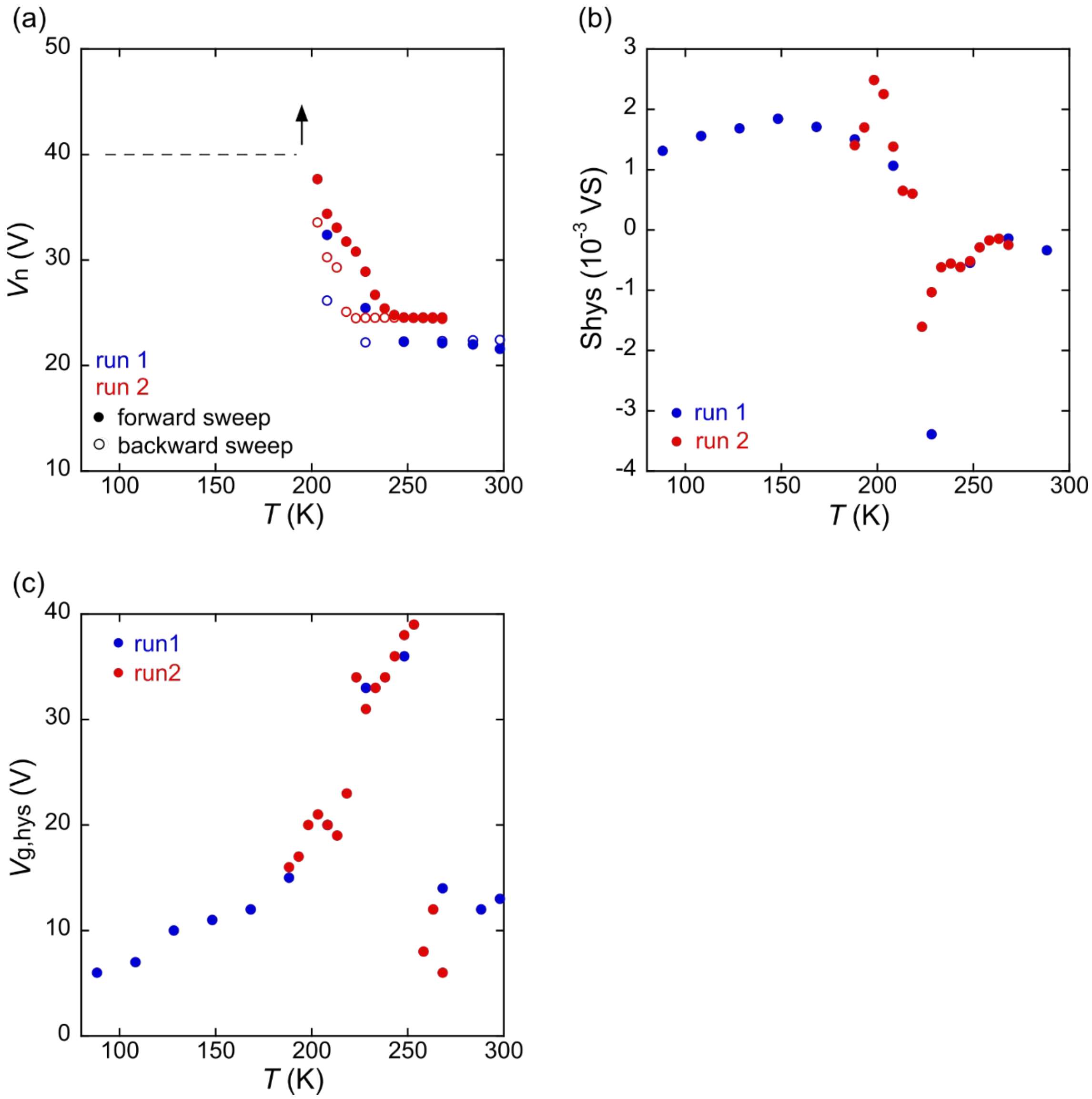

Figure S4. Temperature dependence of parameters derived from the $\sigma - V_g$ curves. (a) Charge neutrality point, V_n. (b) Integrated area enclosed by the upward and downward $\sigma - V_g$ curves, S_{hys}. (c) Averaged gate voltage weighted by the magnitude of the hysteresis, $V_{g,hys}$.

S5. Transport properties of GFETs prepared on hydrophobic substrates

To verify that the observed temperature-dependent behaviors originate from the interfacial water layer, we performed control experiments on hydrophobic substrates. The device fabrication and measurement methods were same as those prepared on hydrophilic substrates. The results for the GFET on hydrophobic substrate are shown in Fig. S5. As shown in Figure S5(a) and (b), $\sigma - V_{\mathrm{g}}$ curves do not exhibit hysteretic behavior and are independent of T except for large $|V_{\mathrm{g}}|$. The σ slightly increased with decreasing T, which is ascribed to the decrease in short-range scattering.[1] Figure S5(c) and (d) show the temperature dependence of charge neutrality point, V_{CNP}, and field-effect mobility, μ_{FE}, at $V_{\mathrm{g}} = 10$ and $30\,\mathrm{V}$. V_{CNP} were independent of T, while μ_{FE} slightly increased with decreasing T. These results further demonstrate that the hydrophilic SiO_2, *i.e.*, the presence of water molecules is responsible for the hysteretic $\sigma - V_{\mathrm{g}}$ curves, the increase in V_{CNP}, and the reduction of μ_{FE}.

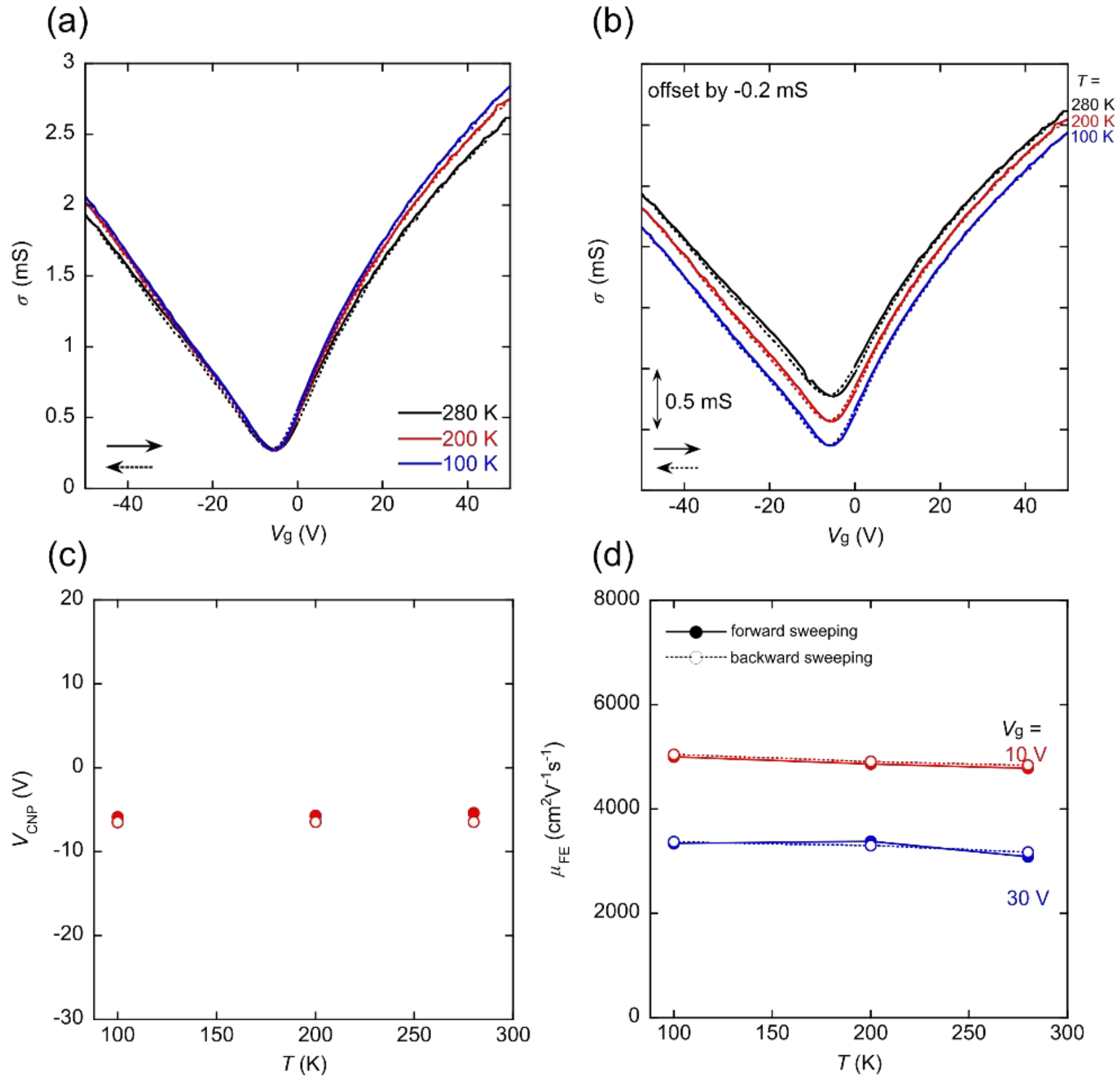

Figure S5. (a) $\sigma - V_{\mathrm{g}}$ curves measured with decreasing temperature. The curves are offset with −0.2 mS in (b). (c) Temperature dependence of charge neutrality point, V_{CNP}. (d) Temperature dependence of field-effect mobility at $V_{\mathrm{g}} =$ 10 and 30 V evaluated from (a).

S6. Reproducibility of ZEFC measurements over multiple cooling–heating cycles

To verify the reproducibility of the inflection points observed in the ZEFC mode, we have measured the $\sigma(T)$ curves in ZEFC mode twice at $V_g = 50$ V using the device #3. (See S8 for the device number.) The results are compared in Fig. S6. Between the two measurements (the 1st and 2nd runs), $\sigma(T)$ curves were measured at various V_gs, which may be the origin of a slight difference in the σ between the 1st and 2nd runs. However, σ shows similar T dependence, which abruptly changes at nearly same temperatures in both measurements (see Fig. S6). In particular, the characteristic transition temperatures to stable solid state and to liquid state remain unchanged, demonstrating that the observed characteristic temperatures are reproducible and are intrinsic features rather than experimental artifacts. These results further support the reliability of the phase diagram proposed in this study.

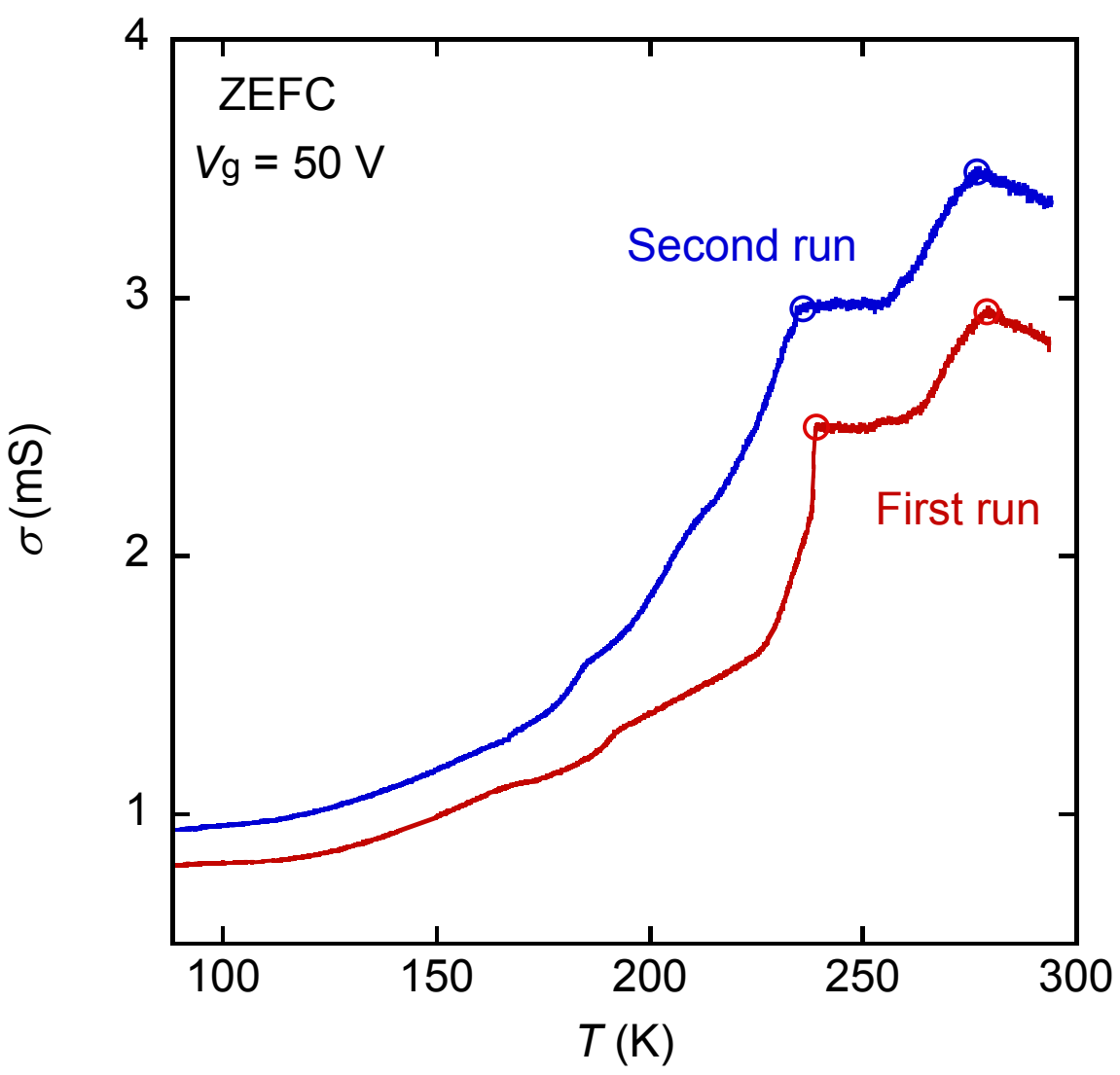

Figure S6. Conductivity of device #3 measured in the ZEFC mode at $V_{\mathrm{g}} = 50\ \mathrm{V}$ twice. The red and blue lines indicate the results for the first and second run. The open circles indicate characteristic transition temperatures to the stable solid state and to the liquid state.

S7. Reproducibility of ZEFC measurements for different heating rates

To evaluate the influence of the heating rate on the inflection point observed in the ZEFC mode, we compare ZEFC measurements at different heating rates (1.2 K min^{-1}, 1.8 K min^{-1}, and 3.5 K min^{-1}), which is called the 1st, 2nd, 3rd run. The results are plotted in Fig. S7. Here, the 2nd and 3rd runs were performed 16 days after the 1st run. Thus, the difference between the 1st run and the other runs might be due to changes over time. However, Fig. S7 indicates that the $\sigma(T)$ curve shifted towards lower temperatures for slower heating. This trend is essential to the nature of the relaxing solid state. The confined interfacial water requires much time to cause structural relaxation and the hydrogen-bond rearrangement at the relaxing solid state. A slower heating rate promotes rearrangement of the hydrogen-bond network of the confined water molecules to reach an equilibrium relaxed solid state at lower temperature.

Though the characteristic temperature may depend on the heating rate, the slope and shape of the $\sigma(T)$ curves are essentially consistent, indicating that the phase transition mechanism is identical. Thus, considering the practical experimental condition, we selected 3.5 K/min as a heating rate.

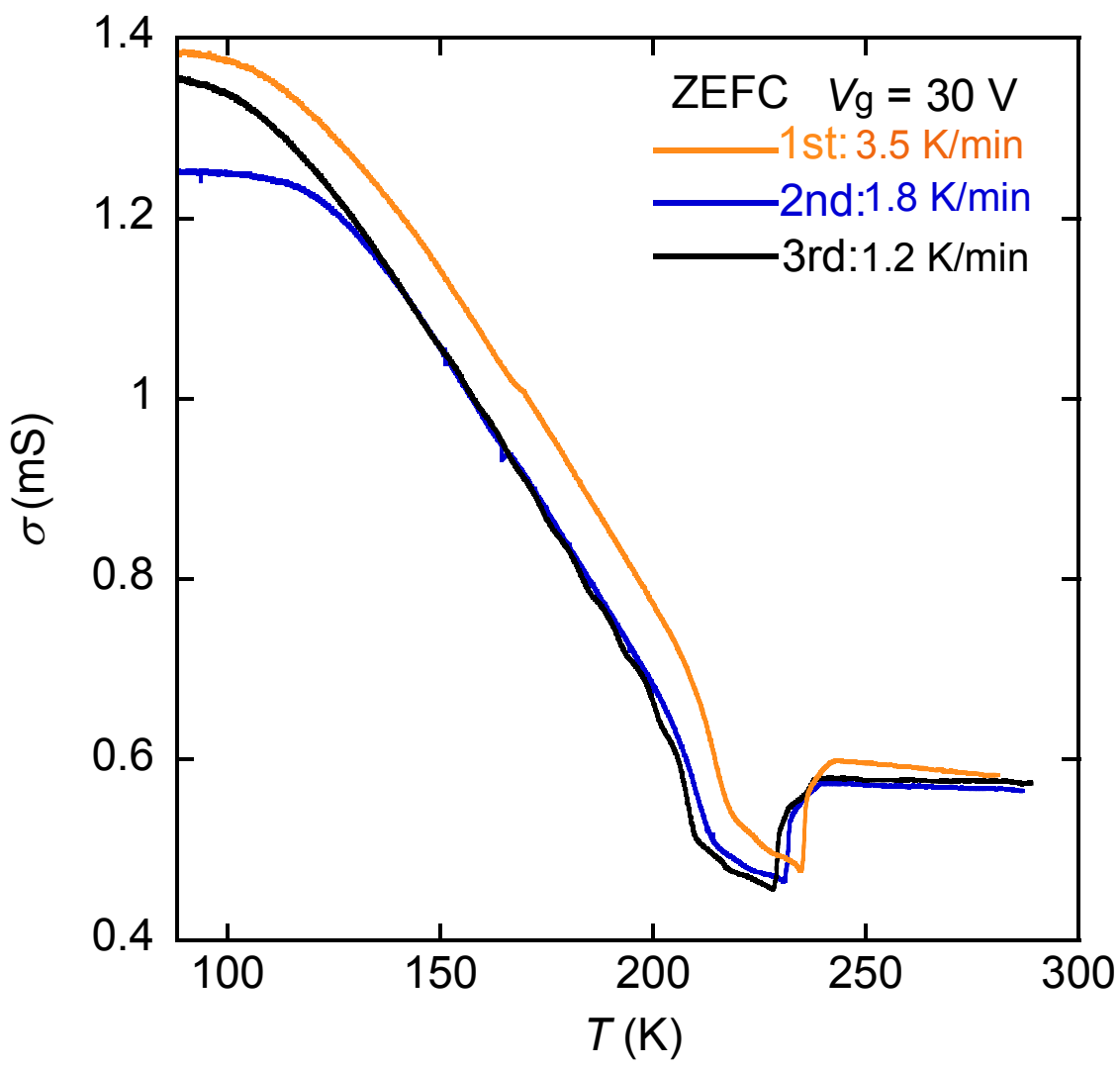

Figure S7. Conductivity measured in the ZEFC mode at $V_{\mathrm{g}} = 30\ \mathrm{V}$ using different heating rate. The heating rates indicated with orange, blue, and black lines are 3.5, 1.8, and 1.2 K/min, respectively.

S8. Reproducibility of $\boldsymbol{\sigma - V_g}$ curve for plural devices

To verify the reproducibility of the results, several devices (device #1, #2, and #3) were fabricated on the hydrophilic SiO_2 under the same conditions as the device (device #0) described in the main text. Figure S8(a-e), Figure S8(f-j) and Figure S8(k-o) were the optical images, $\sigma - V_g$ curves, T dependence of V_{CNP}, T dependence of μ_{FE}, and phase diagrams for the device #1, #2 and #3, respectively. Overall results were reproduced as follows. First, water molecules induce p-type doping, indicating positive V_{CNP} at room temperature. Second, the $\sigma(V_g)$ curve shows significant hysteresis at a high V_g region with decreasing T (see Figure S8(b), (g) and (i)). Third, the V_{CNP} gradually increases as the T decreases (see Figure S8(c), (h) and (m)). Here, the filled and open symbols correspond to V_{CNP} measured at the upward and downward sweeps of V_g, respectively. The discrepancy in V_{CNP} between the two sweeps arises from the hysteretic behavior of the $\sigma - V_g$ curves. Although all devices were fabricated using identical oxygen-plasma treatment conditions and fabrication processes, the values of V_{CNP} at $T = 298$ K was different for device #1 ($= 16.7$ V), device #2 ($= 20.7$ V), device #3 ($= 5.8$ V) and the device #0 ($= 22.0$ V). These differences may be caused by the uncontrollable microscopic environment at the graphene/SiO_2 interface. For example, density of fixed interface charges, density of surface hydroxyl groups, and the

thickness of interfacial water layer, all of which lead to different electronic states. These factors will influence the initial doping level of the graphene, thereby resulting in different V_{CNP}. However, it doesn't affect the overall temperature dependence of transport properties. Fourth, the μ_{FE} decreases abruptly with decreasing T (see Figure S8(d), (i) and (n)). Finally, the phase boundary indicates positive correlation between T and V_{g}. Figure S8(e), (j) and (o) show the regions of V_{g} in which the slope of the transfer curve deviates from that of liquid water at high temperature. These regions define the phase diagram of a water layer which separates the liquid state and the intermediate or solid state. All the devices show similar phase diagrams. These consistent observations demonstrate the reproducibility and reliability of our study.

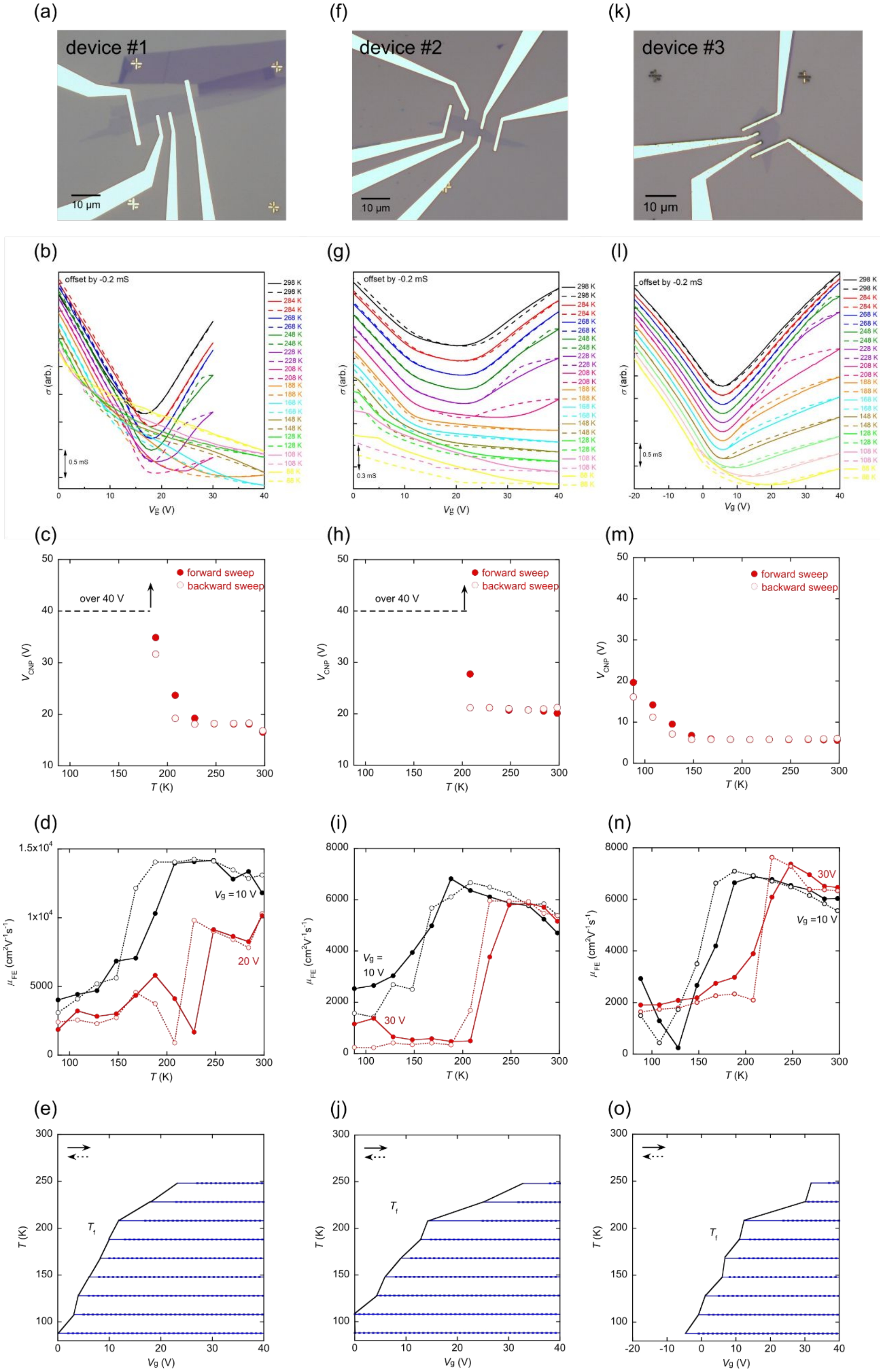
(a)
device #1
10 μm
(f)
device #2
10 μm
(k)
device #3
10 μm
(b)
(g)
(l)
offset by -0.2 mS
σ (arb.)
Vg (V)
(c)
(h)
(m)
forward sweep
backward sweep
over 40 V
VCNP (V)
T (K)
(d)
(i)
(n)
μFE (cm2V-1s-1)
Vg =10 V
20 V
30 V
30V
(e)
(j)
(o)
T (K)
Vg (V)
Tf

Figure S8. Statistical reproducibility of the results. (a-e) for device #1, (f-j) for device #2, and (k-o) for device #3. The optical images in (a), (f), (k), the T dependence of the $\sigma - V_\mathrm{g}$ curves in (b), (g), (l), the T dependence of V_CNP in (c), (h), (m), the T dependence the μ_FE in (d), (i), (n), and the phase diagrams in (e), (j), (o), for each device.

S9. Conductivity at the stable solid state at $V_g =$ 10 and 20 V

As shown in Fig. 3, the conductivity σ reaches its minimum in the stable solid state at $V_g =$ 30 and 40 V. This indicates that the solid with negative polarization P at T_{min} relaxes to a state with polarization $P \sim 0$ before transitioning to the liquid state with positive P. However, we also observe a stable solid state at $V_g =$ 10 and 20 V, in which P remains negative across the entire temperature range. Notably, at $V_g = 10$ V, the σ of the stable solid state is lower than that of the liquid state, whereas the opposite relationship is observed at $V_g =$ 20 V. These experimental facts suggest that the stable solid state in the hole regime is different from that in the electron regime.

Figure S9 schematically illustrates the change in the P under the ZEFC mode. At the RT, P in the liquid state changes from negative to positive with increasing V_g (blue line). In contrast, in the ZEFC mode, P at T_{min} is fixed at the value at $V_g = 0$ V (P_0) and then increases with increasing temperature (red lines). The values of P in the stable solid state are marked with green circles. Based on the experimental results in Figure 3, P at the stable solid state is larger than that at RT for $V_g = 10$ V, but smaller for $V_g = 20$ V.

To explain this, we assume that the system reaches a stable solid state with local $P \sim 0$. This implies that some water molecules relax to a zero-polarization state, while others

remain to have the initial negative polarization P_0. Let the fraction of molecules relaxing to $P\sim 0$ be $1-x(V_\mathrm{g})$, and the fraction remaining at P_0 be $x(V_\mathrm{g})$. The net polarization of the stable solid state is then given by $x(V_\mathrm{g})\,P_0$.

As shown by the black lines in Figure S9, $x=0$ at $V_\mathrm{g}>V_\mathrm{CNP}$, indicating complete relaxation. However, for $V_\mathrm{g}<V_\mathrm{CNP}$, x remains finite and shows only a weak dependence on V_g. We propose that water molecules which cannot relax their orientation are likely trapped near negatively charged impurities, where strong Coulomb interactions prevent reorientation. In this scenario, the value of x is determined by the impurity density, leading to the weak V_g dependence. This scenario of the inhomogeneous relaxation qualitatively explains both the existence of the stable solid state in the hole regime and the distinct σ behavior compared to the liquid state.

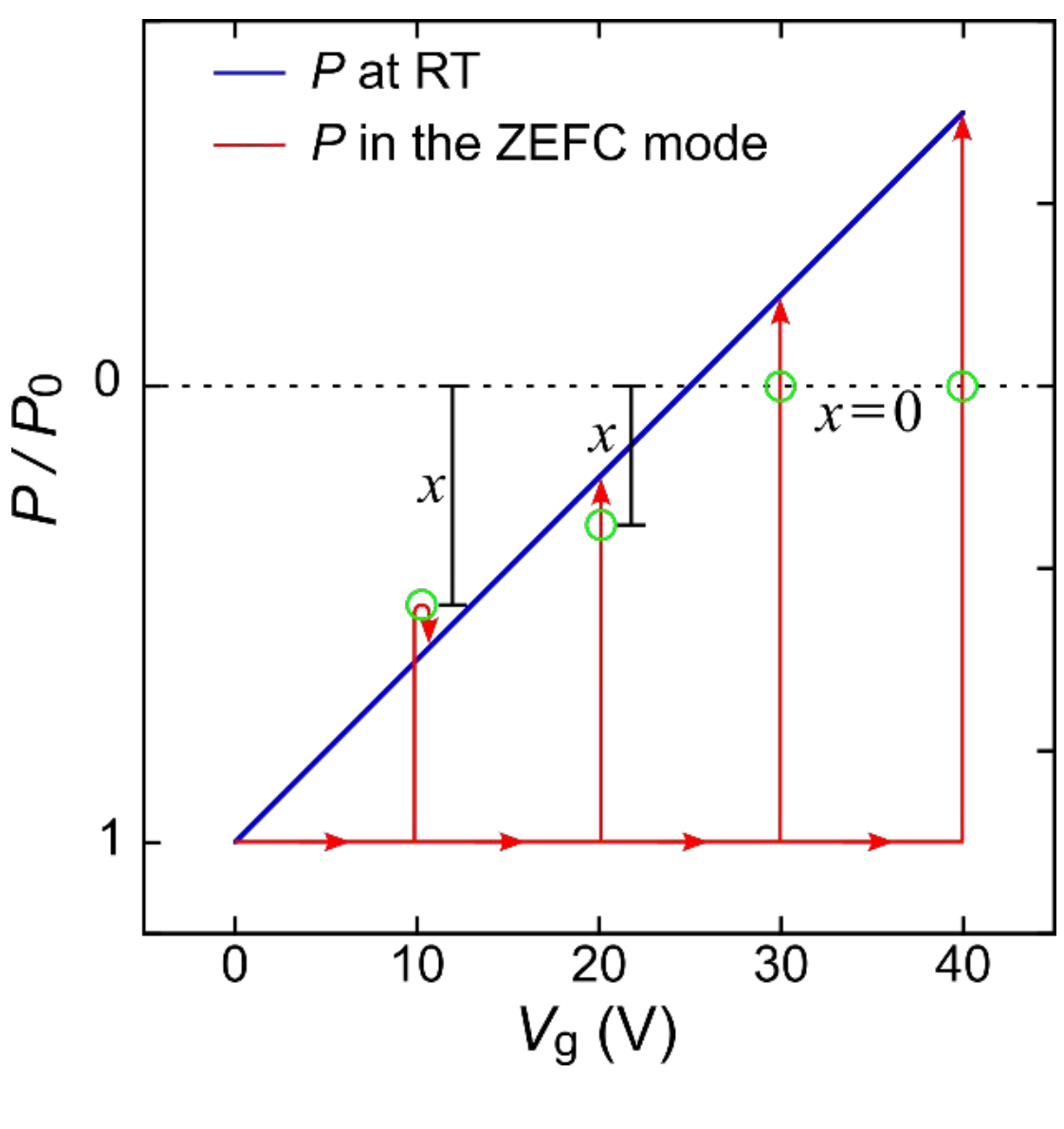

Figure S9 The schematic change in the normalized polarization P/P_0 in the ZEFC mode is indicated with red lines for each V_g. The P is P_0 at T_min and reach to the value at RT indicated with a blue line, via the stable solid states indicated with green circles. The x shows the fraction of water molecules which remain in the state with P_0.

Reference

1. S. V. Morozov, K. S. Novoselov, M. I. Katsnelson, F. Schedin, D. C. Elias, J. A. Jaszczak and A. K. Geim, Giant intrinsic carrier mobilities in graphene and its bilayer. *Phys. Rev. Lett.* **2008,** 100, 016602. DOI: 10.1103/PhysRevLett.100.016602.
